# Investigation into Application of AI and Telemedicine in Rural Communities: A Systematic Literature Review

**DOI:** 10.3390/healthcare13030324

**Published:** 2025-02-04

**Authors:** Kinalyne Perez, Daniela Wisniewski, Arzu Ari, Kim Lee, Cristian Lieneck, Zo Ramamonjiarivelo

**Affiliations:** 1 School of Health Administration, Texas State University, San Marcos, TX 78666, USA; svr30@txstate.edu (K.P.); djw185@txstate.edu (D.W.); kim.lee@txstate.edu (K.L.); zhr3@txstate.edu (Z.R.); 2 College of Health Professions, Texas State University, San Marcos, TX 78666, USA; arzuari@txstate.edu

**Keywords:** artificial intelligence, AI, telemedicine, telehealth, rural healthcare, quality, outcomes

## Abstract

Recent advances in artificial intelligence (AI) and telemedicine are transforming healthcare delivery, particularly in rural and underserved communities. Background/Objectives: The purpose of this systematic review is to explore the use of AI-driven diagnostic tools and telemedicine platforms to identify underlying themes (constructs) in the literature across multiple research studies. Method: The research team conducted an extensive review of studies and articles using multiple research databases that aimed to identify consistent themes and patterns across the literature. Results: Five underlying constructs were identified with regard to the utilization of AI and telemedicine on patient diagnosis in rural communities: (1) Challenges/benefits of AI and telemedicine in rural communities, (2) Integration of telemedicine and AI in diagnosis and patient monitoring, (3) Future considerations of AI and telemedicine in rural communities, (4) Application of AI for accurate and early diagnosis of diseases through various digital tools, and (5) Insights into the future directions and potential innovations in AI and telemedicine specifically geared towards enhancing healthcare delivery in rural communities. Conclusions: While AI technologies offer enhanced diagnostic capabilities by processing vast datasets of medical records, imaging, and patient histories, leading to earlier and more accurate diagnoses, telemedicine acts as a bridge between patients in remote areas and specialized healthcare providers, offering timely access to consultations, follow-up care, and chronic disease management. Therefore, the integration of AI with telemedicine allows for real-time decision support, improving clinical outcomes by providing data-driven insights during virtual consultations. However, challenges remain, including ensuring equitable access to these technologies, addressing digital literacy gaps, and managing the ethical implications of AI-driven decisions. Despite these hurdles, AI and telemedicine hold significant promise in reducing healthcare disparities and advancing the quality of care in rural settings, potentially leading to improved long-term health outcomes for underserved populations.

## 1. Introduction

Access to quality healthcare remains a persistent challenge for rural communities due to geographic isolation, limited healthcare infrastructure, and shortages of medical professionals, all of which contribute to disparities in health outcomes. These challenges have driven innovation in healthcare delivery, with artificial intelligence (AI) and telemedicine emerging as transformative solutions to address these issues [1]. Both technologies have the potential to bridge gaps in care, enhance diagnostic precision, and improve patient outcomes; however, a deeper understanding of their effectiveness and impact on rural populations is needed.

AI has revolutionized various aspects of healthcare by enabling predictive analytics, personalized treatment plans, and streamlined clinical workflows [2]. Machine learning algorithms support early disease detection, while AI-powered decision support systems assist healthcare providers in optimizing the quality of care. Similarly, telemedicine—through virtual consultations, remote monitoring, and telehealth platforms—has expanded access to essential medical services, minimizing the need for travel and ensuring continuity of care for patients in underserved areas [1,3].

Despite their potential, the adoption of AI and telemedicine in rural settings faces several challenges, including limited broadband access, digital literacy barriers, and concerns about data privacy. Furthermore, evidence regarding their direct impact on healthcare quality—such as clinical outcomes, patient satisfaction, and care delivery efficiency—remains fragmented across various studies and contexts [3]. To address these gaps, the research team initiated this review to explore the underlying constructs and themes in the existing literature on the application of AI and telemedicine in rural communities.

## 2. Materials and Methods

### 2.1. Overview and Inclusion Criteria

This systematic review was guided by the Preferred Reporting Items for Systematic Reviews and Meta-Analysis (PRISMA). This research team’s intent was to investigate how AI could be used to improve the quality of care that patients receive, and to determine the potential future applications of AI by analyzing the most recent literature published surrounding this topic. The literature obtained for this article was selected from three research databases (MEDLINE Complete, Complementary Index, and Academic Search Complete), all accessed through the Texas State University Alkek Library’s EBSCOhost search tool. These specific research databases were utilized in this review because of their availability to the researchers via the Texas State University Library’s EBSCOhost online platform, while yielding the highest number of initial number of articles identified that met the search parameters, but was not duplicative of other available databases. The search string was initially formulated using a brainstorming session among the research team members, each providing their input as to the key terms and themes related to the overall topic of AI and rural communities. Using this information, the team conducted several online Google Scholar search queries, identifying the opportunity to include quality of care in the rural healthcare setting as part of the current search.

Focusing specifically on ‘mhealth’ and related publications, the Medical Subject Headings (MeSH) controlled vocabulary thesaurus (National Library of Medicine) was utilized to capture exploding terms for this topic, as well as ‘physical fitness’ and related terminology. Additional research team queries on the EBSCOhost website were conducted to ensure the highest number of published articles germane to the research topic were identified, using the most accessible Boolean search operators to identify the following search string for the study:

[(rural areas or rural communities or rural patients or rural population or remote) AND (telehealth or telemedicine or telemonitoring or telepractice or telenursing or telecare) AND (machine learning or artificial intelligence or deep learning or neural network) AND quality of care AND (diagnosis or diagnosing or diagnostics or assessment or screening)]

For an article to be included in this review, the article had to be published between 1 January 2020–31 December 2024 on the research database. The database queries carried out by the research team occurred from 2 to 28 February 2024. The 2024 ending search date range was automatically selected by EBSCO, the host website, and therefore, picked the most recent publications during the team’s search period. Other EBSCOhost search criteria included: Full Text, Peer Reviewed, and English Only. Full text articles are complete articles found online, as opposed to just the summary or abstract. To be considered peer reviewed, an article must undergo evaluation by experts in the field of study. Editorials, government reports, letters to editors, or other non-reviewed studies were also not included in the search.

Articles within the systematic review had to focus on or address the use of AI and/or telemedicine within rural communities, either as the primary research topic, or at least address how AI and/or telemedicine could improve quality of care. The team carried out a search for the most pertinent literature available to assess the use of AI and telemedicine in rural communities, both currently and potential future innovations. The information in the study did not include human subjects (secondary data sources), with all the literature being publicly available and published. If any study found by the team focused on any individual research subject(s), they were not identifiable to the team. Therefore, an institutional review board (IRB) review was not required, and obtaining consent was not necessary.

### 2.2. Exclusion Process

**Figure 1:**
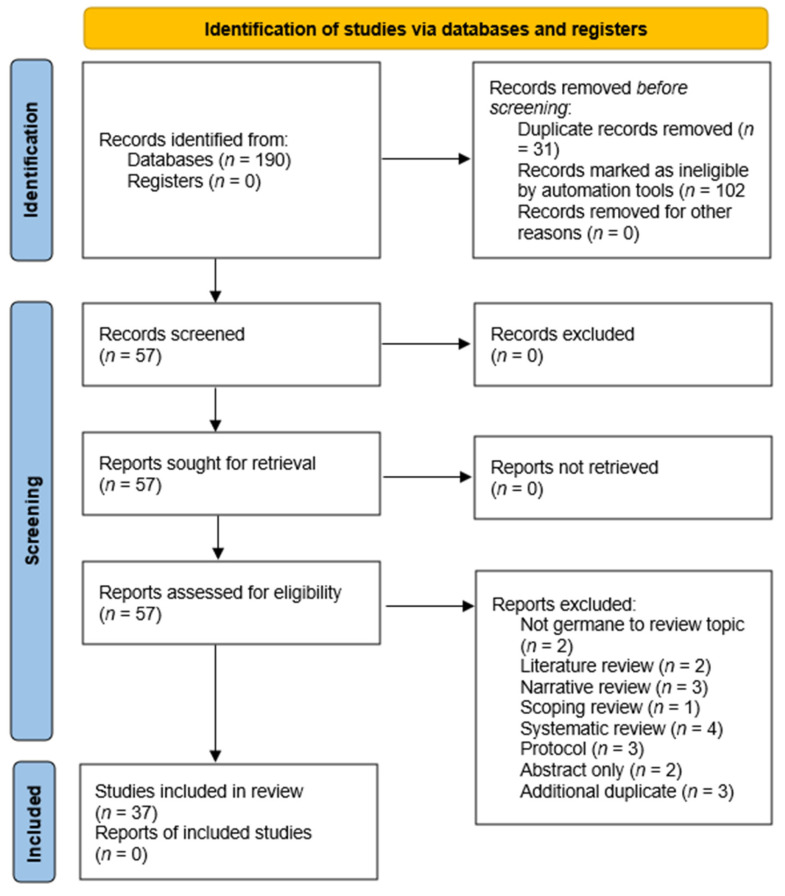
Preferred reporting items for systematic reviews and meta-analysis (PRISMA) figure demonstrating the study selection process.

Figure 1 demonstrates the article exclusion process for the review. Figure 1 starts with our initial research database results and ends with our final literature sample (n = 57). Our initial search on EBSCOhost found 190 articles that fit our primary criteria; however, 31 articles were automatically removed from the search results by the EBSCOhost search engine, identified as duplicate articles. Additional articles were then removed from the search results because they were marked as ineligible by automation tools. Of these remaining articles in the EBSCOhost search results, 102 articles were excluded due to automation tools:

- 28 articles were removed for not being available in full text,
- 52 articles were removed for not being published in peer reviewed journals/outlets,
- 21 articles were removed because they were not published within the review’s prescribed date range.

The research team accessed all 57 articles for retrieval in full-text format for further analysis. Each article was reviewed by at least two of the research team’s members. Each member was assigned roughly 20 articles to review and determine if the articles were relevant to our literature review, and to see if any articles had slipped through our automated screening tools. Table 1 shows how all 57 articles were split up and assigned to each team member to review.

Upon completion of the full-text review, the research team collectively decided to exclude an additional 17 articles from the review. Two articles were identified as not germane to the current study’s review topic. Two articles were only available to the research team via the EBSCOhost platform as abstract only and three articles were study protocols. Three additional articles were identified as duplicates. Finally, 10 additional articles were removed from the review process for being classified by the review team as either a literature review, systematic review, narrative review, or a scoping review (Figure 1).

## 3. Results

Guided by the PRISMA protocols, the research team presented their initial article review findings by identifying each study’s population, intervention, control method(s), outcome(s), and study design as applicable. Further review of the articles by the research team involved assessing the articles for specific AI and telemedicine application, specifically in a rural health environment. Often, such articles included the delivery of care utilizing these resources in an ambulatory care/outpatient industry segment. Initial, individual article findings are presented in Table 2.

**Figure 2:**
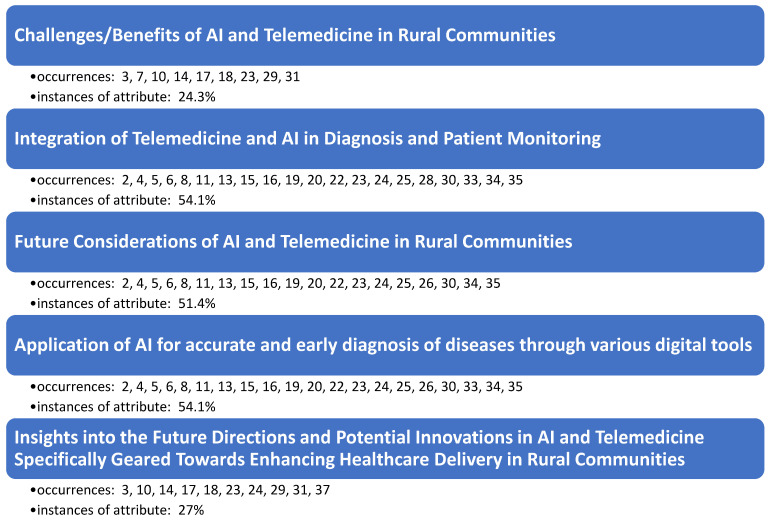
Occurrences of underlying themes as observed in the literature.

Results of the research team’s consensus meetings demonstrate five main themes (constructs) as identified in the literature to support the use of AI in rural communities. Findings are not mutually exclusive to only one specific theme, as several articles supported more than one construct upon review (Figure 2).

The systematic literature review findings reveal a significant focus on the integration and future implications of AI and telemedicine in rural communities, with these topics comprising the majority of the discussion. The construct “Challenges/Benefits of AI and Telemedicine in Rural Communities” was noted in 24.3% of the instances, underscoring both the potential advantages, such as improved access to healthcare, and the barriers, including technological limitations and resistance to adoption in rural settings. The most frequently addressed constructs, “Integration of Telemedicine and AI in Diagnosis and Patient Monitoring” and “Application of AI for Accurate and Early Diagnosis of Diseases through Various Digital Tools” independently accounted for 54.1% of the instances. This highlights the critical role of AI and telemedicine in enhancing diagnostic accuracy and enabling continuous patient monitoring in these areas. Finally, “Future Considerations of AI and Telemedicine in Rural Communities” appeared in 54.1% of the instances, emphasizing the importance of ongoing technological advancements and policy developments to fully realize the potential benefits of AI and telemedicine in rural healthcare.

## 4. Discussion

### 4.1. Challenges/Benefits of AI and Telemedicine in Rural Communities

Integrating AI and telemedicine into healthcare systems in rural or underdeveloped areas can drastically alter the healthcare landscape, though this transition is fraught with both significant challenges and profound benefits. Several barriers hinder the widespread adoption and effectiveness of telemedicine and AI. On one hand, regions like rural India face numerous practical obstacles, including insufficient technological infrastructure, intermittent power supply, limited internet access, and a lack of trained healthcare professionals, all of which are essential for the effective deployment of AI and telemedicine [18]. Limited access to high-speed internet and outdated technology infrastructure can impede the seamless delivery of virtual healthcare services. Additionally, the high costs associated with implementing advanced technologies can be prohibitive, limiting the spread of beneficial innovations [18]. Hypothesized barriers to telemedicine use include doctors’ beliefs that it may have little benefit, reduce revenue, and negatively impact their standing within the community [18]. Also, healthcare providers may need specialized training to effectively utilize telemedicine tools and AI technologies. Addressing these training needs is crucial to ensure that rural healthcare professionals can use the full potential of these innovations. Furthermore, data privacy and security present additional barriers to the adoption of telemedicine and AI in rural healthcare settings.

Benefits of integrating AI and telemedicine are substantial and multifaceted. One of the most notable advantages is the increased access to healthcare services, overcoming geographical barriers that often hinder rural residents from receiving timely medical attention. For instance, AI can greatly enhance the quality of care through improved diagnostic accuracy and personalized treatment plans, even in remote locations where specialized medical expertise is scarce [18]. Telemedicine extends healthcare’s reach, allowing patients in isolated areas to receive timely consultations, thus overcoming geographical barriers that traditionally impeded access to care [3,18]. Furthermore, telemedicine platforms can predict health status with maximum accuracy and facilitate continuous monitoring and management of chronic diseases, improving patient outcomes and reducing hospital visits [7,14,17]. Interactive telemedicine systems may include real-time communication between patients and healthcare providers, reduce travel time and ensure timely triage [10]. By combining telemedicine and AI, rural healthcare systems can leverage technology to bridge the gap in healthcare access and provide quality medical care to populations that have historically been underserved.

The deployment of AI in diagnostics and patient management systems can reduce the workload on the limited number of healthcare providers in rural settings, increasing efficiency and allowing more patients to be treated effectively [18]. AI applications in health data analysis can also predict outbreak patterns, which is crucial for preventive healthcare in underdeveloped regions.

Telemedicine and AI also offer economic benefits by reducing the need for physical infrastructure and decreasing travel times for both patients and healthcare providers. This not only lowers healthcare delivery costs but also improves the utilization of healthcare resources. Additionally, innovative applications like drone technology can facilitate the rapid delivery of medical supplies to remote areas, further bridging the gap in healthcare accessibility.

While the implementation of AI and telemedicine in rural or underdeveloped areas faces considerable challenges, the potential to significantly improve healthcare accessibility, quality, and efficiency holds immense promise. Overcoming these challenges requires targeted investments in infrastructure, training, and policy adjustments to ensure that the benefits of these technologies can be fully realized, ultimately leading to more equitable health outcomes [7,18].

### 4.2. Integration of Telemedicine and AI in Diagnosis and Patient Monitoring

#### 4.2.1. Advancing Remote Diagnostics Through AI and Telemedicine

The integration of AI and telemedicine has significantly enhanced the capacity for remote diagnostics and early disease prediction. Technologies such as AI-powered platforms for stroke diagnosis and diabetic retinopathy screening enable early identification of critical health issues, particularly in underserved regions where access to specialized care is limited [2,4,5]. For example, AI models have been shown to accurately detect retinal diseases using fundus photography and deep learning, which reduces the reliance on in-person examinations [15]. These advancements not only facilitate earlier interventions but also improve patient outcomes by providing timely and accurate diagnoses. As these tools evolve, their capacity to predict and monitor diseases remotely continues to bridge the gap in healthcare accessibility [2].

#### 4.2.2. Wearable Technologies and IoT for Continuous Monitoring

The integration of wearable devices and Internet of Things (IoT) frameworks in telemedicine has revolutionized patient monitoring by enabling continuous data collection and real-time analysis [2]. Wearables such as smart socks for phlebopathic conditions and cardiac monitoring implants provide clinicians with actionable insights into patient health without requiring frequent in-person visits [5]. These technologies are particularly beneficial for managing chronic conditions, as they allow for early detection of complications and reduce the burden on healthcare systems. Moreover, the use of IoT facilitates seamless connectivity between devices and medical platforms, ensuring efficient data flow and enhancing the accuracy of remote monitoring systems [2].

#### 4.2.3. Precision and Automation in Patient Monitoring Systems

Machine learning has become integral to telemedicine by improving the precision and reliability of patient monitoring systems [1]. Automated tools such as video-based activity recognition for Parkinson’s disease and neonatal sound quality assessment reduce the need for manual observations while delivering consistent results. These innovations also enable scalable monitoring solutions, particularly in clinical trials and large-scale health programs [5,13]. By leveraging advanced algorithms, telemedicine systems can provide precise diagnoses and insights, empowering healthcare providers to optimize care delivery [5,13]. This emphasis on automation not only enhances diagnostic accuracy but also alleviates the workload for healthcare professionals.

#### 4.2.4. Scalability and Cost-Effectiveness of AI-Driven Solutions

The scalability and cost-effectiveness of AI-powered telemedicine solutions are pivotal in expanding healthcare access to diverse and resource-limited settings. Autonomous systems such as LumineticsCore for diabetic retinopathy have demonstrated their effectiveness in increasing screening rates while maintaining high diagnostic accuracy [16]. These systems are designed to operate seamlessly across varied practice environments, making them suitable for rural and urban healthcare facilities alike. Furthermore, the integration of AI with multimodal imaging, such as OCT angiography, supports complex diagnostic workflows and enhances the overall quality of care. As telemedicine technologies continue to advance, their affordability and adaptability will remain crucial for addressing global healthcare disparities [15].

### 4.3. Future Considerations of AI and Telemedicine in Rural Communities

AI and telemedicine are reshaping healthcare delivery, offering innovative solutions to address the persistent challenges faced by rural communities [3,10]. These technologies are advancing personalized care, improving diagnostics, facilitating remote monitoring, and reducing the time and cost associated with accessing healthcare services. As these tools continue to evolve, their potential for enhancing healthcare delivery in underserved areas becomes increasingly evident.

Key advancements include the development of a Real-Time AI and Internet of Medical Things (IoMT) Engine for Mobile Health Edge Computing. This technology uses Sparse Auto-Encoders Deep Learning and Group Method of Data Handling (GMDH) neural networks to predict diseases such as stroke, achieving high diagnostic accuracy and supporting remote medical care in smart living environments [2]. Similarly, AI-powered video-based activity recognition is being developed for automated motor assessments of conditions like Parkinson’s disease, enabling clinicians to remotely evaluate patient actions with precision [20].

The integration of IoT infrastructure in healthcare is also expanding, with innovative tools such as smart socks for detecting phlebopathic conditions and intelligent systems for low-cost remote disease detection and treatment [5]. For neonatal care, automated models that assess heart and lung sounds via telehealth applications are enhancing the quality of care by enabling accurate, remote estimations of heart and breathing rates, which are critical for preventing neonatal mortality and morbidity [11].

Machine learning models are also driving breakthroughs in predictive analytics, such as a frailty phenotype prediction model that achieved a sensitivity of 82.70% and specificity of 71.09%, underscoring the potential for AI to guide targeted interventions [22]. Additionally, programs like the Indian Health Service-Joslin Vision Network Teleophthalmology Program are leveraging AI and telemedicine to enhance compliance with diabetic retinopathy standards among underserved populations, including American Indians and Alaska Natives [3,16,17,19,25].

Emerging technologies continue to show promise for rural healthcare. For instance, robotic-assisted examinations, developed through initiatives like the ProteCT project during the COVID-19 pandemic, demonstrate how automation can address healthcare provider shortages [13,33]. Remote monitoring tools, such as the CardioMEMS device for hemodynamic status tracking, are enhancing chronic disease management by enabling real-time health monitoring and reducing hospitalizations [33].

Future applications of AI and telemedicine hold the potential to further transform rural healthcare [3,17,18]. Advances in imaging techniques, generative adversarial networks for enhancing portable ultrasound imaging, and tele-ophthalmology platforms are expanding diagnostic capabilities. These technologies, coupled with continuous innovation, will likely enable earlier interventions, more personalized care, and improved health outcomes for rural populations [10,18].

An integration of AI and telemedicine technologies offers a pathway to reducing healthcare disparities in rural communities. By enhancing diagnostic accuracy, enabling real-time monitoring, and facilitating access to care, these advancements are poised to revolutionize rural healthcare delivery, ultimately improving patient outcomes and healthcare efficiency. Continued investment in research, infrastructure, and equitable implementation will be critical to realizing the full potential of these transformative technologies.

### 4.4. Application of AI for Accurate and Early Diagnosis of Diseases Through Various Digital Tools

Some articles proposed the combined use of Internet of Medical Things (IoMT) mobile health app and AI to better serve patients residing in remote and hard to reach areas. More specifically, some studies combined IoT with AI for a more efficient data storage and management through a medical decision support system [8], for predicting some serious health issues [8], including the predictions of stroke, heart attack, brain strokes, and sudden fall [2], the telemonitoring and screening patients suffering from phlebopathic disease using smart sock technology [5], the evaluation of the sound quality of the hearts and lungs of newborn babies, using binary classification model, for future telehealth applications [11], the combination of telemedicine and AI to examine retinopathy of prematurity (ROP), and the use of teleophthalmology program for surveillance and management of diabetic retinopathy among American Indian and Alaska Native populations [23]. The main purpose of the program was to increase diabetic retinopathy screening rate [23]. While this article commented that teleophthalmology is more cost-effective than conventional dilated retinal examination to detect diabetic retinopathy, the authors mentioned that the use of AI systems for diabetic retinopathy diagnosis are not validated at the American Telemedicine Association Category 3 program, and the performance of these AI systems has not been studied among Amarian Indian and Alaska Native populations. The authors suggested that AI systems offer opportunities for the program to improve the performance of the Reading Center, as well as the triage of the patients [23]. Regardless, Boyle, Vignarajan, and Saha [26] embedded AI components (image quality and disease detection algorithm) into a telehealth platform to detect diabetic retinopathy among indigenous Australian communities residing in remote areas. Higher diabetic retinopathy detection rate was reported at the patient level than at the eye level [25].

Another study developed a third-party system to collect and administer remote monitoring RM (data) from implantable cardiac electronic devices (CIEDS) and enable a real-time and automatic centralized of CIEDS data from multiple hospitals [6]. While AI was not used in this study [6], the authors recommended that combining RM data resources with AI may further enhance the prediction of lead malfunctions, battery depletion, and arrhythmia [6]. 

The use of Human Activity Recognition, in terms of video recordings of body movements, and AI has also helped researchers predicting the severity of Parkinson’s disease based on the Movement Disorder Society Parkinson’s Disease Rating Scale (MDS-UPDRS) [4]. AI based on machine learning techniques with the combination of wireless wearable sensor devices (Internet of Things) was also used to determine patients’ physical frailty phenotypes (slowness, weakness, and exhaustion) [22]. AI, based on two-stage generative adversarial network, was used to obtain the optimal image quality of hand-held/portable ultrasound devices [26]. 

Some studies in our review used telemedicine without AI. Those studies are mostly concerned with COVID-19 (51 and 54). A telemedical diagnostic system equipped with a robotic arm was used to examine emergency patients, more specifically those who contracted infectious diseases such as COVID-19 [33]. The authors concluded that the acceptance of the new technology was high among physicians, and robotic telemedical devices have the potential to complement healthcare beyond COVID-19 [33]. Labetoulle at al.’s article [34] discussed the use of telescreening for remote ocular surface assessment as a solution to protect eye-doctors from contracting infectious disease, especially during COVID-19 pandemic. Ophthalmologists, among other eye doctors, have a higher risk of contracting COVID-19 because they need to closely approach the patients’ faces for effective eye exam and COVID-19 patients might need to see an ophthalmologist if SARS-CoV-2 causes conjunctival hyperemia [34]. However, the authors concluded that telescreening does not have the capability to replace standard ophthalmic examination, particularly for ocular surface disease. However, there is still hope of having effective telescreening thanks to groundbreaking research in high-technology imaging. 

### 4.5. Insights into the Future Directions and Potential Innovations in AI and Telemedicine Specifically Geared Towards Enhancing Healthcare Delivery in Rural Communities

Future directions in the integration of artificial intelligence and telehealth highlight the potential to transform the healthcare landscape through innovative technologies. One promising example is the development of learning-based disease prediction models that utilize telemedicine and AI to screen for and diagnose eye and ear, nose, and throat (ENT) diseases [3]. These models have demonstrated the capability to remotely screen patients, monitor their conditions, prioritize healthcare resources, enhance disease prediction and diagnosis, and enable clinicians to provide more personalized treatment plans [3,24]. Additionally, deep learning models offer opportunities for greater patient involvement in their own healthcare, such as allowing individuals to capture medical images at home and securely share them with their clinicians for further analysis [10].

However, the integration of AI and telehealth must also address critical ethical and regulatory considerations to ensure these technologies are implemented effectively and responsibly. Key policy recommendations include providing comprehensive training for nurses, clinicians, and other healthcare professionals to enhance their technological literacy and ensure they can confidently utilize these tools [3]. Equally important is educating patients on how to properly use at-home healthcare technologies to promote engagement and accuracy in remote care. Furthermore, it is essential to address challenges related to patient privacy and informed consent by establishing clear guidelines that safeguard sensitive health data and ensure transparency in AI-driven decision-making. Another critical consideration is the careful design and rigorous testing of AI algorithms to minimize biases that could lead to disparities in healthcare delivery and outcomes. Ensuring fairness, accountability, and equity in AI applications will be crucial for maximizing their benefits while maintaining trust and ethical standards in healthcare.

Including underrepresented communities is important to ensure an equity of outcome for those patient population that are being served [10]. Another policy recommendation is to standardize how data collected from IoT’s are formatted, how data are collected, who owns the data, and how that data are used and protected [14,29]. Standardization of data ensures that the data that is collected is reliable and consistent across different healthcare systems and allows IOT devices to share information. Those in healthcare also have an ethical duty to ensure that patients’ personal data are safe and secure; therefore, a risk management policy should be created to ensure that a patient’s data are safe [17].

Future telemedicine platforms have the potential to incorporate AI and IoT devices to enhance patient care, increase accessibility, and decrease costs. Potential innovations include AI-driven diagnostic tools [24]. An AI-driven diagnostic tool is a software application that uses AI algorithms to assist in the diagnosis of medical conditions. Many AI-driven algorithms that are currently being developed for rural communities involve the analysis of medical images to detect and diagnosis patients with diseases. IoT devices can also be utilized to collect large amounts of continuous data for AI algorithms to analyze and make clinical diagnoses [24]. The idea of these AI-driven diagnostic tools is to enable nonclinical specialists to be able to diagnose patients with diseases in rural areas where there are not a lot of clinical specialists. Doing so would decrease the health inequality gaps that rural patients face by increasing access to healthcare. However, employers must provide training programs to educate non-clinical healthcare workers on how to use these AI-driven diagnostic tools efficiently and accurately [16,17]. AI-driven diagnostic tools can be pushed even further. When enough advancements in AI-driven diagnostic tools have been made, these tools can be used by patients themselves to diagnosis themselves [10]. If this is to be achieved, then patients too must be trained in how to diagnosis themselves. However, these tools must be patient, friendly and easy to use. Future directions should also include the creation and implementation of security measures for the data that is collected from patients, transferred, and stored for patients [29]. IoT devices capture large amounts of private medical information that are protected by HIPPA. To avoid legal issues, strict security and privacy standards as well as a top-notch security system should be put in place for the safe storage of information and safe communication among IoT devices and the place where the data will be stored [24]. If such standards are not put in place, third parties could obtain access to that data and perform unauthorized activities with that data [24].

## 5. Conclusions

### 5.1. Summary of Findings

With the increased access to care in hard-to-reach rural communities, thanks to telemedicine, and the improved accuracy in diagnostic thanks to the use of AI, the authors review extant peer-reviewed articles published between January 1, 2020, and December 31, 2024 to synthesize the findings regarding the impact of the AI and telemedicine on patient diagnoses and quality of care in rural areas. Three constructs emerged from the review of the 40 articles that met the inclusion criteria, namely the challenges and benefits of the use of AI and telemedicine in rural communities, the integration of telemedicine and AI in diagnosis and patient monitoring, and the future considerations of AI and telemedicine in rural communities.

### 5.2. Review Contributions

Some of the barriers to the widespread adoption of AI and telemedicine in rural areas consist of the lack of resources and infrastructure that these technologies require, high cost, and shortage of specialized workforce, including healthcare providers, who are expected to use these technologies. Other barriers to adoption consist of concerns regarding data privacy and protection as well as whether technology is easy to use. Regardless, telemedicine and AI provide some healthcare benefits. For instance, telemedicine has been found to expand timely communication with healthcare providers and patients residing in remote areas; telemedicine eliminates travel costs and lengthy travel times. Also, the use of AI to assist healthcare providers with accurate diagnosis can increase quality of care because with accurate diagnosis, patients receive the appropriate treatment for their diseases, increasing the probability of positive health outcomes.

Regarding the use of AI and telemedicine to enhance diagnostic accuracy and continuity in patient monitoring, this review found that AI and telemedicine have been used to predict stroke, heart attacks, physical frailty and sudden fall, monitor implantable cardio-electronic devices, and monitor phlebopathic patients, as well as those diagnosed with type II diabetes, especially to prevent blindness due to diabetic retinopathy and age-related macular degeneration. These technologies have also been used to detect and diagnose cancer.

Based on our review, both telemedicine and AI have the potential to improve access to patient diagnosis and therefore quality of care in rural healthcare organizations. There is an emerging trend combining telemedicine and AI to further improve access to and quality of care, with respect to accurate disease prediction and diagnosis, as well as health outcomes. However, nationwide regulations, protocols, and guidelines are needed to standardize telehealth and telemedicine services, ensure ethical practices, mitigate bias that may be embedded in healthcare data when used in AI.

### 5.3. Review Limitations

While studying the impact of AI on patient diagnoses and quality of care has profound implications for access to care and access to technology, it is essential to recognize and address the limitations inherent in studies such as sample bias, measurement bias, and ethical concerns during the investigation of this complex research topic. Additionally, a formal quality assessment of each article identified in the review could provide deeper insights and enhance the robustness of the findings, in an attempt to future reduce bias and/or assess rigor in future research.

### 5.4. Future Directions and Possible Applications

Future directions discussed include the development of technology that could change the healthcare landscape with the incorporation of AI technology and telehealth. One such example is the creation of a learning-based disease prediction model that uses telemedicine and AI to screen for and diagnosis eye and ENT diseases [3]. This model has revealed the potential to remotely screen patients, track patient conditions, prioritize resources, improve disease predictions, diagnosis, and classification, and allow clinicians to provide personalized treatment to patients [3,24]. Other deep learning models have the potential to allow patients to become more involved in their healthcare, such as by allowing patients to take images at home and send them to their clinicians [10].

The research team identified the integration of the Internet of Things (IoT) and AI with telemedicine as a relevant concept that may hold future potential for enhancing healthcare delivery. IoT-enabled devices can provide real-time patient data, while AI algorithms can analyze this information to support timely and accurate clinical decision-making in remote settings, supporting the premise of this review. This convergence presents a promising opportunity for future research to explore its impact on improving diagnostic precision, patient monitoring, and overall healthcare accessibility, particularly in underserved communities.

When discussing the future direction of incorporating AI with telehealth, one must also consider policy recommendations to ensure that these innovative technologies are used ethically and to their fullest potential. Policy recommendations that should be considered include having nurses, clinicians, and other healthcare providers be trained in technological literacy [3]. On top of this, patients should also be trained in how to use these at home technologies. Another policy recommendation is to ensure that algorithms are carefully designed and tested to mitigate any amount of bias. Including underrepresented communities is important to ensure an equity of outcome for those patient population that are being served [10]. Another policy recommendation is to standardize how data collected from IOT’s are formatted, how data are collected, who owns the data, and how that data are used and protected [14,29]. Standardization of data ensures that the data that is collected is reliable and consistent across different healthcare systems and allows IOT devices to share information. Those in healthcare also have an ethical duty to ensure that patients’ personal data are safe and secure; therefore, a risk management policy should be created to ensure that a patient’s data are safe [17]. In conclusion, further research into the infrastructure, political landscape, and socioeconomic factors that influence the adoption of artificial intelligence and telemedicine in diverse rural settings is essential to fully understand and address the challenges and opportunities these technologies present.

## Figures and Tables

**Table 1:** Reviewer assignment of the initial database search findings (full article review).

Article Assignment	Reviewer 1	Reviewer 2	Reviewer 3	Reviewer 4	Reviewer 5	Reviewer 6
Articles 1–20	X	X	X	X	X	X
Articles 21–40			X	X		X
Articles 41–57					X	X

**Table 2:** Summary of findings (n = 37).

Article Number	Article Title and Author Name(s)	Population/Intervention		Control/Outcomes	Summary of Findings
[1]	Harnessing Artificial Intelligence: Strategies for Mental Health Nurses in Optimizing Psychiatric Patient Care Nashwan, S. et al. (2023)	The study focused on psychiatric patients and mental health nurses. The researchers utilized databases such as PubMed, IEEE Xplore, ScienceDirect, and PsycINFO to gather relevant literature. They used keywords including “Artificial Intelligence”, “Mental Health Nursing”, “Psychiatric Care”, and “Machine Learning” to conduct their search. AI methods like Natural Language Processing (NLP) and Machine Learning (ML) were employed to predict and detect depression early, forecast suicide attempts, and enhance the prediction of eating disorder symptoms.		Studies have explored various wearable AI devices that monitor physiological and emotional indicators in different mental illnesses. AI applications were examined for their ability to track user symptoms and manage episodes of anxiety and depression. Digitally assisted nursing observations in acute psychiatric units led to improved experiences for both patients and staff. AI demonstrated effectiveness in detecting and diagnosing various mental health conditions. Chatbots were successfully used to diagnose and screen patients for anxiety, depression, PTSD, and suicide risk. Studies reported positive outcomes from using chatbots as assessment tools, including high accuracy, increased engagement, early diagnosis, and even prevention of mortality.	AI in Mental Health Care: ○ AI applications are increasingly facilitating mental health care by enabling early detection of mental health conditions, supporting clinical decision-making, assessing risks, and ensuring treatment adherence. ○ AI has revolutionized remote patient monitoring and telehealth services. ○ The integration of AI in healthcare necessitates prioritizing ethical considerations, data privacy, and security. AI health technologies are being incorporated into clinical simulation laboratories, featuring tools such as humanoid robots, cyborgs, and face tracker software.
[2]	Mobile AI Stroke Health App: A Novel Mobile Intelligent Edge Computing Engine based on Deep Learning Models for Stroke Prediction – Research and Industry Perspective Elbagoury, B. et al. (2021)	This study focuses on patients that are at a high risk for developing stroke disease. The goal of the study is to provide a remote intelligence diagnosis engine for stroke prediction and diagnosis for patient emergency cases using mobile health technology. The app detects strokes by using EMG signals from sparse auto coders from 20 classes of EMG datasets via a mobile app and wearable IOTs.		The control in this study is the use of traditional diagnosis during the time of a stroke event via emergent (ER) triage and care. A high performance rate of stroke diagnosis was achieved using sparse auto-encoders, reaching a 98% accuracy rate in stroke prediction.	Expanding Specialist Care: ○ Mobile App with AI predicting stroke utilizing EMG signals and comparing and using EMG dataset for prediction can be implemented for real-life scientific implementation by larger tech companies such as Dell. ○ Expanded medical dataset and features using mobile AI engine for refined prediction and diagnosis suggested as next research study. ○ Application of mobile AI Stroke Health App for adaptation for smart health living at home for early prediction of stroke events and for routine monitoring of patients at risk.
[3]	Use of Telemedicine and Artificial Intelligence in Eye and ENT: A Boon for Developing Countries Upadhyaya, P. et al. (2022)	The population being addressed in this study consist of citizens and patients in rural, impoverished, and hard-to-reach areas of Nepal. The intervention in this study uses telehealth and AI by using deep learning that is administered by a team of IT Professionals and healthcare specialists at a hub center that is connected to a remote location where non-specialist health workers called Community Medicine Assistance (CMA’s) operate equipment to screen and diagnose eye and ENT diseases. The CMA’s were trained in operating the equipment and diagnosing Eye and ENT diseases. The use of deep learning-based prediction models in Diabetic Retinopathy and Glaucoma added specialty services to telehealth. The model was used to predict patients with risk of diabetic retinopathy and glaucoma.		The control group in this study used telehealth without the addition of AI or deep learning for prediction and diagnostic care. There was also no care provided for high-risk patients with eye diseases. The study achieved a sensitivity of 98.57%, a specificity of 92.97%, an Area under the Curve of 0.988, and found the Clinical Utility to be excellent (0.9154) for measuring and predicting diabetic retinopathy. The functional outcome of this study was that blindness was being averted through early diagnoses in hard-to-reach areas. The glaucoma prediction model achieved a sensitivity of 92.74%, and specificity of 96.49%, and a clinical utility of 0.849. Overall, the outcome was greater access to those that live in hard-to-reach populations in poor, rural areas.	Mitigation of Disease Burden: ○ Telemedicine combined with deep learning modeling AI was found to mitigate the burden of disease in a developing nation of Nepal. This shows relevance for extending access and quality of care to other rural, remote, and poor populations in other developing countries. Expanding Specialist Care: ○ Telemedicine combined with AI expanded the use of skilled specialists at major hubs with non-specialist workers in remote areas – further reducing burden on skilled specialists by using the non-specialist workers with AI and telemedicine. Demand for services still outweighed the capability of telemedicine, AI, and provision of care.
[4]	Video-Based Activity Recognition for Automated Motor Assessment of Parkinson’s Disease Sarapata, G. et al. (2023)	The study focused on patients with Parkison’s disease across multiple centers within the US and UK. One intervention used in this study was the Human Activity Recognition (HAR) framework to classify tasks performed by patients with Parkinson’s disease. Another intervention used in this study was the use of AI to train a deep learning model. AI trained the learning model by analyzing body joint locations using an open source pose estimation library. The model was then evaluated using the data within the large multi-site dataset of the MDS-UPDRS Part 3 data recordings. The final intervention used in this study was prediction modeling, which was based upon frame-by-frame video analysis, allowing the model to recognize multiple activities.		The control in this study was an assessment that used the Movement Disorder Society Unified PD Rating Scale (MDS-UPDRS) without the use of body joint markers, deep learning, and AI. A vision-based Human Activity Recognition (HAR) framework capable of dynamically classifying tasks performed motor assessment tasks performed by patients with Parkinson’s disease. A deep learning model using estimated body joint locations was trained to distinguish between 8 MDS-UPDRS items corresponding to 15 activity classes with 96.51% accuracy.	Remote Motor Assessment: ○ This technology and model can help achieve remote, asynchronous Parkinson’s Disease (PD) motor assessment at scale leading to larger-scale remote trials and patient-driven programming, non-invasive neuromodulation devices that can assist with rehabilitation and medical management.
[5]	Smart Sock-Based Machine Learning Models for Phlebopathic Patient Screening D’Angelantonio, E. et al. (2022)	This study focused on patients with phlebopathic diseases. The sample population consisted of 24 participants, 13 healthy and 11 suffering from chronic peripheral vascular disease and diabetic foot. The Timed Up and Go (TUG) test was carried out by patients during medical examinations at outpatient facilities, with the presence of physicians, while the healthy participants performed the same test at the university research laboratory. The TUG test was instrumented with pressure and inertial sensors integrated in the SISTINE sensor sock, worn on the right foot. The sensorized sock was connected to a microcontroller board, the Arduino Nano BLE Sense, which integrates an internal Low Energy Bluetooth module used to connect the device to the Android Application developed to acquire data. The mobile application was developed through MIT AppInventor tool and installed on a Wiko Y81 (Android 10) smartphone.		The study focuses on chronic diseases such as chronic vascular diseases (CVD) of the lower limbs and the diabetic foot, which are included in the phlebopathic diseases. Data were viewed from any device connected to the internet, allowing physicians and technical operators to monitor patients and visualize the trend of the collected data. The best classifier obtained showed a particularly superior performance, with an accuracy of 95.83%, high precision, recall, and a F1-Score equal to 0.95. The wearable system will not only be dedicated to data collection but will also be an active interface from which the user can receive information and warnings on his health status. The proposed framework is a viable candidate for screening phlebopathic patients and for their early diagnosis.	Data Collection and Machine Learning: ○ Wearable technologies enable the collection of large amounts of data, enabling the development of machine learning models to identify diseases, complications, or monitor treatment effects. Assisting in Patient Evaluation: ○ The use of technology can assist in the evaluation of the patient’s disease condition, giving back quantitative parameters to the physician.
[6]	A mutually communicable external system resource in remote monitoring for cardiovascular implantable electronic devices Hasumi, E. et al. (2023)	This study focuses on patients with implantable cardiac electronic devices (CIEDs). In total, 1136 patients had an implantable cardiac pacemaker (ICP), 1050 had an ICD or cardiac resynchronization therapy defibrillator (CRT-D), and 56 had an implantable loop recorder (ILR). The intervention was an in real-time automatic centralized system of CIED information from six hospitals.		The control in this study was the remote monitoring data of CIED patients within each hospital. The remote monitoring data were then integrated using the ORFICE-Local software. The centralized remote monitoring system could be a new platform that promotes the use of device data as big data. The data aggregated from multiple centers could be used to develop an AI algorithm for detecting CIED abnormalities in the future. The combination of third-party remote monitoring data resources and AI may have favorable compatibility. If AI algorithms are applied to the timely generated big data of CIEDs, then many clinical parameters or events may be predicted, including lead malfunctions, premature battery depletion, and arrhythmia.	Benefits of Combining AI and Remote Monitoring: ○ The combination of third-party remote monitoring data and AI offers significant benefits. Feasibility of Centralized Monitoring: ○ There is demonstrated feasibility in centralizing a system for CIED remote monitoring data. Impact on Clinical Practice: ○ The information collected from a CIED database across multiple hospitals in real-time could greatly enhance individual clinical daily practices.
[7]	A computer-aided pre-diagnosis system for health prediction based on personal health data Preity, R. et al. (2023)	The population in this study consists of people in rural areas that do not have premium healthcare facilities. The intervention has a total of 10 efficient features that were extracted for accurate prediction. The model uses six widely used, high-powered popular classifiers: Naïve Bayes (NB), logistic regression, Support Vector Machine (SVM), k-Nearest Neighbor (k-NN), Decision Trees (DT), and random forest. The system uses weight, medical data, age, and gender as input features from the patient. The combination of third-party remote monitoring data resources and AI may have favorable compatibility. If AI algorithms are applied to the timely generated big data of CIEDs, then many clinical parameters or events may be predicted, including lead malfunctions, premature battery depletion, and arrhythmia.		The control is a system that uses a Graphical User Interface (GUI) to predict an individual’s health status based on basic health parameters. The study found that the support vector machine classifier outperforms other methods with maximum classification accuracy of 100%. The GUI allows users to enter their vital health data and predict their health status. The predictive model could be less efficient in diagnosing mental health fitness due to limited real-world clinical health data and health features.	AI in Healthcare: Early Diagnosis and Chronic Diseases: Early diagnosis can play a crucial role in preventing chronic diseases. The paper highlights that 80% of the population aged 50 and above, with a history of high blood pressure, cholesterol, blood sugar, or being overweight, are more likely to develop chronic diseases. Predictive Model and GUI: The designed user-friendly GUI can predict health status with maximum accuracy of 100%. Limitations in Mental Health Diagnosis: The predictive model could be less efficient in diagnosing mental health fitness due to limited real-world clinical health data and health features.
[8]	An intelligent IoT based healthcare system using fuzzy neural networks Preity, R. et al. (2020)	The study focused on remote patients located in South Punjab. The intervention is a system that uses a medical decision support system to process patient data and provide a low-cost solution for remote areas. The system can detect serious health issues and provide treatment by contacting nearby hospitals.		The control is a generic model for IoT based healthcare system that identifies key components with an end-to-end IoT healthcare system. The proposed system collects body temperature, respiration rate, and heartbeats and observes the patient’s body movements. The outcome was that the average response time of queries responded to by CDSS was quite short compared to the response time of queries responded to by physicians. The proposed system is an efficient low-cost solution for people of remote areas.	Accuracy and Cost Savings: Implementing IoT based patient monitoring devices shows reasonable accuracy and cost saving. Decision-Making Systems: Fuzzy Logic System is an excellent choice for decision-making systems and provides lightweight devices and software components. Impact of Additional Sensors: Adding more sensors will certainly improve patient data collection and therefore improve diagnosis.
[9]	The impact of COVID-19 on chronic pain: multidimensional clustering reveals deep insights into spinal cord stimulation patients Berger, S. et al. (2023)	The study had a population size of 70 subjects. Of these subjects, 61.4% female, 60 ± 9.4 years mean age, 15.1 ± 10.7 years mean duration of chronic pain, and 171.8 ± 58.7 days mean ENVISION study follow-up duration. All 70 subjects were candidates for SCS treatment of chronic pain in the low back (98.6%), unilateral leg (41.4%), and/or bilateral leg (42.9%). The intervention studied the subjects’ responses to social changes, increased community stress, and reduced access to care. Evaluations included in-clinic questionnaires, self-reported ratings, physiological measurements, and voice recordings.		The control consists of daily self-reported ecological momentary assessments (EMAs) and weekly voice recordings that were compared between the baseline period and pandemic period. The gap period between baseline and COVID-19 was defined as a buffer for COVID-19 spread and pandemic awareness. Outcome measures include pain, standardized questionnaire scores, mood/emotion, voice, sleep quality/quantity, cardiac function, wrist worn actigraphy, medication use, spinal fluoroscopic imaging, SCS parameters/usage, functional fitness, treatment satisfaction, and quality-of-life reports. Three distinct patient responses (sub-cohorts) were revealed: worsened pain, reduced activities, or improved quality-of-life.	Benefits of AI and Digital Technology: The study found that AI techniques, when combined with multiple data collection mechanisms, can provide deeper insights into patient experiences. AI simplifies complex data and aids in real-time physician decision-making to improve outcomes beyond just pain intensity. The introduction of digital technology allowed for the inclusion and assessment of novel, more naturalistic, and richer data streams not typically available via traditional research studies or patient care delivery. Impact of Digital Health Tracking: COVID-19 highlights the importance of continuous digital health tracking and quantification, particularly in chronic pain monitoring and SCS treatment.
[10]	Telemedicine and delivery of ophthalmic care in rural and remote communities: Drawing from Australian experience Kiburg, K. et al. (2022)	The population in this study consisted of Aboriginal and Torres Strait Islander Communities in rural and remote Western Australia who needed ophthalmic eye care. The intervention in this study consisted of different forms of telemedicine in ophthalmology, each with unique logistical and technological requirements. The use of telemedicine and AI is backed by leading professional bodies, with RANZCO and Optometry Australia identifying that AI can improve diagnosis accuracy, lead to better patient outcomes, and is likely to play a major role in eye care in the future.		The study looked at several different models such as a semi-automatic model, a fully automated model, current human assessment model, current annual assessment model, real-time teleophthalmolgy video consultations, and deep learning systems. The outcome of this study was that the semi-automatic model was found to be the most cost-effective (USD 62 per patient per year) followed by the fully automated model (USD 66 per patient per year). The current human assessment model was the least cost-effective (USD 77 per patient per year). Savings of 20% of the current annual screening model were estimated due to expected growth in diabetic retinopathy patients. Real-time teleophthalmology video consultations have been a significant factor in patient satisfaction in rural Western Australia. On-call telehealth for visiting optometry in regional Western Australia has also improved patient access to eye care. Lastly, the integration of telemedicine and AI into outreach eye care has shown benefits in enhancing patient care.	Benefits and Impact of AI and Telehealth: The study found that the use of AI and telehealth/teleconsultations increased remote patient access to care and provided economic benefits to the delivery of care. Advances in technology provide a promising future for increased access to screening and assessment of eye diseases, especially in disadvantaged communities in rural and remote Australia. AI has been introduced in ophthalmology, particularly in disease screening, to increase referrals, reduce travel time, and ensure timely triage. Technological and Systemic Considerations: Interactive telemedicine systems may include real-time communication between patients, clinicians, and specialists via video, requiring coordination of clinician and patient availability and increased technological demands. Challenges and Barriers: Barriers to improving the disparity in disease burden include reduced access to screening and treatment, distrust of the medical system, and a lack of culturally appropriate services and non-clinical support services. Despite the existence of telemedicine models, a lack of culturally appropriate care has been identified, requiring the inclusion of Indigenous people in all stages of program design and implementation.
[11]	Neonatal Heart and Lung Sound Quality Assessment for Robust Heart and Breathing Rate Estimation for Telehealth Applications Grooby, E. et al. (2022)	The population in this study consisted of 76 babies within Monash Newborn Australia, with 17 being preterm and 59 being full term. The intervention used was a digital stethoscope such as the CliniCloud to monitor the babies’ hearts and their breathing rates. The study recorded 88 samples, each being 10 s-long chest sounds from the babies.		The control consisted of six annotators that independently assessed the signal quality, number of detectable beats, and breathing periods from recordings. Five quality segments were created: 1 (very poor) only noise, no detectable heart beats; 2 (poor) mostly noise with some detectable heart sounds; 3 (borderline) heard heart sound but contaminated by noise, difficult to interpret; 4 (good) easily heard heart sound with weak noise, interpretable; 5 (excellent) clear heart sound with little to no noise. The outcome was that the HR Peak + Segmentation method performed best on low quality signals with a median absolute error of 6.7 bpm and interquartile range of 12 bpm. Performing a Wilcoxon signed-rank test, this method was significantly better on low quality sounds than HR Schmidt et al. The HR and BR estimated from high quality sounds resulted in significantly less median absolute error compared to those from low quality sounds. The model distinguished high- and low-quality recordings in the test set with 96% specificity, 81% sensitivity, 93% accuracy for heart sounds and 86% specificity, 69% sensitivity, and 82% accuracy for lung sounds.	Opportunities and Benefits: Newborn telehealth presents an opportunity for increased access, reliability, and quality of healthcare for diagnosis and prognosis. Signal Quality and Remote Monitoring: Ensuring the signal quality of digital stethoscopes can enable patients in remote areas or those with limitations accessing healthcare facilities to digitally monitor their health in conjunction with their provider.
[12]	Development of 5G-based Remote Ultrasound Education: Current Status and Future Trends Ma, J. et al. (2023)	The population in this study consisted of medical practitioners/physicians delivering ultrasounds. The intervention consisted of the use of 5G technology to provide remote ultrasound education to enhance the quality of ultrasound education through focusing on two aspects: The development of ultrasound medical teaching resources in both in-person and online courses, based upon the learner’s preference (via the National Telemedicine and Internet Medicine Center), and the building of a network, three-tiered infrastructure including a (1) tertiary hospital with technical capabilities to construct 5G and AI-integrated remote models, which would be able to transcend boundaries and disparities between regions and hospitals (1st tier); (2) secondary network encompassing the equipment outside the ultrasound department in the hospital and other departments’ US equipment in secondary hospitals (mainly responsible for implementing project and serving as a bridge between 1st tier and 3rd tier); (3) primary institutions at the grassroots level, relying on remote consultations and AI support to collect and transmit US data.		The control group consists of face-to-face, in classroom courses, and training on ultrasound expertise. The outcome for the first intervention was that the survey results revealed that students from three groups (first group = remote learners, second group = on-site learners, and third group = combination approach of remote and on-site learning) gained knowledge and their research capabilities had improved. 88.6% (39/44) students thought they improved significantly. However, there was no statistically significant difference between the three groups (*p* > 0.05). For the second intervention, the construction of the laboratories for phases 1 and 2 were completed. However, phase 3 (grassroot coverage of remote ultrasound consultation) has not yet been complete.	Implications and Innovations: The implementation of a two-way assessment system, where both learners and instructors evaluate each other concurrently, is an innovative approach that enhances the improvement process for both learners’ outcomes and the quality of instruction at the same time. Results showed that learning outcomes are significant; however, there was no substantial difference in satisfaction between online and in-person learning. This indicates that a dynamic medical education program can be effectively achieved through innovative methods. Future Implications: There is the opportunity to expand and develop markets for more widespread and advanced ultrasound education by using a three-tiered network approach with 5G and AI technologies for improved diagnosis, consultation, and education.
[13]	Current advancements in therapeutic approaches in orthopedic surgery: a review of recent trends Liang, W. et al. (2024)	The population consists of orthopedic surgery patients. The interventions include regenerative medicine techniques such as stem cell therapy and platelet-rich plasma (PRP) injections. The study involves the application of robotic-assisted surgery to enhance surgical precision and outcomes. Personalized medicine approaches are used to tailor treatments to individual patient needs and characteristics. Telemedicine is utilized to provide remote consultations and follow-up care for orthopedic patients. Remote patient monitoring systems are employed to track patient progress and manage recovery from a distance. AI and machine learning technologies are integrated into orthopedic practice to improve diagnostic accuracy, treatment planning, and patient outcomes.		The control interventions include arthroscopy surgical techniques, which use a small camera known as an arthroscope to view and treat joint issues. Joint replacement techniques, specifically arthroplasty, are employed for replacing damaged joints. Spinal fusion procedures are used to join two or more vertebrae in the spine to stabilize it. Advancements in therapeutic approaches, such as regenerative medicine, robotic-assisted surgery, and personalized medicine, have significantly improved patient outcomes, reduced recovery times, and enhanced the overall quality of care in orthopedic surgery. The integration of AI and machine learning, along with advancements in regenerative medicine, telemedicine, and 3D printing, is shaping the future of orthopedic care, leading to more effective and personalized treatments.	AI and Machine Learning (ML): AI and ML have been recognized as beneficial for both clinicians and patients due to their cost-effectiveness, improved patient outcomes, and enhanced quality of care in orthopedic surgery. Robotics: Robotics in orthopedic surgery have contributed to faster recovery and improved patient outcomes by assisting surgeons in conducting procedures with greater precision, safety, and accuracy. AI Guidance: AI can provide guidance to surgeons during orthopedic procedures, thereby decreasing the likelihood of mistakes and improving surgical accuracy.
[14]	Eldo-Care: EEG with Kinect Sensor-Based Telehealthcare for the Disabled and the Elderly Das, S. et al. (2023)	The population consists of the elderly and individuals with psycho-neurological conditions. The intervention consists of EEG sensors that were placed on the scalp to monitor brain activity and look for signs of mental illness, seizure disorders, cognitive strain, among other neurological abnormalities. A filter technique, the Chebyshev filter, eliminated electronic noise and purified data. Kinect Sensors were placed on 25 anatomical sites (body joints) to measure movement during the experiment. After data collection, noise reduction, and feature extraction, classification occurred in which convolutional neural networks were used for categorization of mental activity, physical activity, and identification of the problematic region of the brain, which could/can be targeted for use of transfer learning to enhance rehabilitation results.		The control was conventional treatment and rehabilitation without the use of EEG, Kinect data sensors, and transfer learning. Results showed that convolutional neural networks along with the transfer learning method performed classification with an accuracy of 95% and higher.	Advantages and Potential Benefits This technique shows promise and potential high impact of helping to refine the effectiveness and efficiency of traditional neuro-rehabilitation techniques by offering a “biofeedback” and “real-time” attenuator of connecting with the most critical brain areas most directly related to movement and specific targeted function. This technique shows promise in reducing stress and improving memory in patients during the rehabilitation process, and shows promise in cognitive rehabilitation. This technique will help track a patient’s mental and brain function during the rehabilitation process. This technique could also potentially help patients with other physiological impairments obtain better outcomes. Ultimately, this technique shows implications for better quality of life for patients.
[15]	Multimodal Imaging, Tele-Education, and Telemedicine in Retinopathy of Prematurity Almadhi, N. et al. (2022)	The target population in this study was patients with Retinopathy of Prematurity (ROP). This study employed the use of multimodal digital imaging that used fundus fluorescein angiography (FA), optical coherence tomography (OCT), and OCT angiography (OCTA) to obtain high-resolution images that can be used in telemedicine and tele-education with diagnosing ROP.		The control was a conventional diagnosis of ROP without use of multimodal imagining techniques. The outcome was that with a combination of approaches (FA, OCT, OCTA) and AI modeling for screening and diagnosis there were more capabilities and possibilities to diagnose ROP.	Increased Access and Accuracy Multimodal digital imaging contributes to, and AI capabilities lend to, screening and diagnosis of ROP via telemedicine platforms. These are implications for increased access of remote patients to care and increased screening and accuracy of diagnosis of eye disease(s).
[16]	Determinants for scalable adoption of autonomous AI in the detection of diabetic eye disease: key best practices learned through collection of real-world data Goldstein, J. et al. (2023)	The target population within this study are adult patients with diabetes, particularly those at risk of diabetic retinopathy and macular edema, across diverse geographic locations and demographic backgrounds. These include patients in health systems with varying sizes, staffing resources, finances, and demographics, encompassing the Midwest, Southwest, Northeast, and West Coast of the United States. This intervention includes the deployment of the first fully autonomous AI system, LumineticsCoreTM (formerly IDx-DR), for the detection of diabetic retinopathy without the need for a specialist physician overread at the point-of-care. The intervention includes training for operators and healthcare providers, integration into clinical workflows, and the use of key performance indicators (KPIs) to measure the number of eye exams performed per month.		The primary outcomes assessed were the scalable adoption and utilization of the autonomous AI system, measured as the attainment number of exams per month using the autonomous AI system against targets set for each health center. Secondary outcomes include improvement in quality of care, reduction in health disparities, efficiency in clinical workflow, and staff satisfaction. Over a 12-month period, the aggregated number of diabetes-related eye exams per month increased significantly, indicating the success of the AI system in improving access and quality of care for diabetic retinopathy detection.	Technology and Implementation: LumineticsCore is an autonomous AI system that diagnoses diabetic retinopathy without requiring specialist overread at the point of care. The system received U.S. FDA De Novo authorization, marking a significant advancement in diagnosing diabetic retinopathy directly at the point-of-care. Technology and Implementation: LumineticsCore is an autonomous AI system that diagnoses diabetic retinopathy without requiring specialists at the point of care. The LumineticsCore is the first fully autonomous AI system, LumineticsCoreTM, received U.S. FDA De Novo authorization for diagnosing diabetic retinopathy without specialist physician overread at the point-of-care. Deployment and Adoption: Since the launch of LumineticsCore, the number of diabetes-related eye exams has increased. The deployment of this AI system across four diverse health systems highlights its potential for scalable adoption. Success Factors: The successful adoption of fully autonomous AI in healthcare settings is influenced by three primary determinants: Inclusion of Executive and Clinical Champions, Underlining Health Center Resources, and Clinical workflows that contemplate patient identification, LumineticsCore Exam Capture, and Provider Consultation, and Timely Referral Triage.
[17]	Economic Evaluation of combined population-based screening for multiple blindness-causing eye diseases in China: a cost-effectiveness analysis Hanruo, L. et al. (2023)	This study focused on the Chinese population aged 50 years and older, encompassing individuals in both rural and urban settings, targeted for population-based screening for multiple major blindness-causing eye diseases including age-related macular degeneration, glaucoma, diabetic retinopathy, cataracts, and pathological myopia. Three different screening delivery options: non-telemedicine (face-to-face) screening, AI-enabled telemedicine screening, and non-AI telemedicine screening. The interventions were evaluated for their cost-effectiveness and cost–utility from a societal perspective, considering both direct and indirect costs.		Primary outcomes include incremental cost–utility ratios (ICURs) based on quality-adjusted life years (QALYs) and incremental cost-effectiveness ratios (ICERs) in terms of cost per blindness year avoided. Secondary outcomes focus on healthcare utilization, patient satisfaction, clinician revenue, public hospital system and patient health expenditures, patient health outcomes, diagnostic and treatment knowledge of specific health conditions, and overall receipt of healthcare services.	Benefits of Telemedicine and AI: Telemedicine enhances coverage, accessibility, quality, efficiency, and affordability of care, especially in ophthalmology where image-based diagnosis is crucial. AI in telemedicine improves diagnostic accuracy and service coverage. Cost-Effectiveness of AI Screening: AI telemedicine screening for eye diseases is cost-effective in both rural and urban areas, showing higher efficiency compared to non-AI telemedicine and face-to-face screenings. Impact on Quality and Accessibility: AI-enabled telemedicine promotes equity in eye health, improves care quality, and is economically advantageous, demonstrating its effectiveness in increasing accessibility and diagnostic precision.
[18]	Achieving health equity through healthcare technology: Perspective from India Prakamya Gupta et al. (2023).	The study’s population encompasses the Indian population facing health inequities, particularly marginalized and rural communities lacking access to healthcare services. The intervention has healthcare technologies including indigenous medical devices, diagnostic products, telemedicine, AI, and drone technology aimed at integrating rural needs, improving health outcomes, patient safety, and healthcare quality.		The outcomes include improvement in health outcomes, reduction in out-of-pocket expenditure, increased patient safety, and enhancement of healthcare quality and experience. The outcomes also include increased accessibility, affordability, availability, and awareness, thus reducing healthcare disparities.	Impact on Quality and Accessibility The introduction of indigenous medical devices and diagnostic products as part of healthcare technologies aimed at promoting health equity by making healthcare more accessible and affordable, thus potentially improving diagnosis and treatment outcomes. The adoption of telemedicine and digital health technology to overcome barriers such as geographical isolation, socioeconomic status, and language, which have traditionally limited access to healthcare services. This includes the use of telemedicine platforms like eSanjeevani, which connect patients in rural areas with doctors in urban centers, enhancing the availability and accessibility of healthcare services. The application of AI in healthcare, which includes patient triaging, disease forecasting, population-based disease screening, and decision support applications, indicating AI’s role in improving diagnosis accuracy, patient care, and management efficiency. AI technologies are being used to bridge health inequalities by providing data-driven insights and decision-support tools to healthcare providers, particularly in primary care settings. The overall discussion on how these technologies collectively contribute to advancing rural health equity by addressing the key factors of accessibility, affordability, and awareness, demonstrating a multi-faceted approach to improving diagnosis, quality of care, and overall health outcomes in underserved populations. How AI can overcome logistical issues The use of drones to deliver medical supplies to remote or hard-to-reach areas, showcasing how technology can overcome logistical barriers to healthcare access and delivery, especially in emergency situations or areas with poor infrastructure.
[19]	Telemedicine for Age-Related Macular Degeneration Christopher J. et al. (2020)	The study’s population are patients with Age-Related Macular Degeneration (AMD), a leading cause of vision loss in the United States. The study discusses the challenges and opportunities for telemedicine in the management of AMD. The intervention comprises the implementation of telemedicine strategies for AMD, which include remote screening, monitoring, and management using digital imaging and AI applications. The interventions explored vary from feasibility studies to clinical experiences with telemedicine systems for AMD.		The outcomes comprised the evaluation of feasibility, sensitivity, specificity, and clinical agreement of telemedicine strategies compared to traditional clinical examinations. Outcomes include the effectiveness of telemedicine in detecting characteristic lesions of AMD, making clinical recommendations, and managing established AMD through remote monitoring systems. Additionally, the potential for AI systems to accurately grade images for features of AMD is considered.	Gap Between FDA Approval and Investigators Despite there being no FDA-approved AI system for AMD, there is growing interest in these systems for image processing and interpretation in AMD. Researchers are training deep learning algorithms to identify features of AMD on color fundus photographs and OCT images. Challenge between AI and scientist scope of knowledge The difficulty in confirming fundus photos for AMD diagnosis, given that fluorescein angiography and the conventional clinical examination are the gold standards. However, telemedicine approaches have been investigated with differing degrees of accuracy and agreement using both stereoscopic and monoscopic images. The divergent views of the value of population screening for AMD and the difficulties the illness presents for telemedicine, such as the need for more sophisticated imaging modalities than those used in telehealth systems for diabetic retinopathy. Quality and Reliability of AI Clinical experiences with adopting ocular telehealth programs for AMD, such as in situ studies and randomized controlled trials, have explored remote interpretation of auxiliary data and imaging for AMD management without causing major delays in therapy or bad effects.
[20]	A digital companion (eCARE-PD platform) for people living with Parkinson’s: A co-design study Grosjean et al. (2022)	The population includes people living with Parkinson’s disease (PwP), including a varied sample in terms of gender, stage of Parkinson’s disease, age, time since diagnosis, and living area (urban/rural). This population reflects a diversity that ensures the eCARE-PD platform can meet a wide range of needs and preferences. The intervention process was used to create the eCARE-PD platform, a digital companion that is part of the intervention. This platform seeks to provide tailored assistance for PwP self-management practices, integrating digital health communication and perhaps improved by a machine learning algorithm-based recommender system. The goal of the intervention is to enhance teamwork among healthcare providers and offer individualized care guidelines and strategies.		The eCARE-PD platform now incorporates essential design concepts that were determined via the co-design process and are based on the requirements and expectations of users. Among the results is the creation of a platform that is collaborative, meaningful, responsive, adaptive, instructive, interactive, and adjustable. Iterations upon iterations of the design process are intended to provide a socially acceptable technology that facilitates at-home self-care and produces customized digital health communication, hence improving PwP’s quality of life and self-management.	ECARE-PD goals and workings By offering individualized support and enhancing communication with the healthcare team, the eCARE-PD platform seeks to enhance integrated care models for individuals with Parkinson’s disease. This could result in improved self-management techniques and a higher quality of life. The study’s co-design methodology makes sure the digital companion satisfies the unique requirements and expectations of its potential users, which is essential for the technology’s efficacy and social acceptance. The eCARE-PD platform is designed to be an interactive, meaningful, tailored, empathic, and socially acceptable technology, reflecting the collective solutions identified through the co-design process. AI Implementation Benefits The use of AI to improve personalized care by choosing customized care recommendations and recommending personalized care plans that are most relevant to the users is suggested by the possible integration of a machine learning algorithm within the platform for the purpose of developing a recommender system. The incorporation of Parkinson’s disease survivors’ experiences into the co-design process underscores the significance of user-centered design in the creation of digital health tools that facilitate self-care within the framework of an integrated care paradigm.
[21]	Telepsychiatry and Outpatient Department Services Laxmi Naresh et al. (2020)	The population consists of patients in need of psychiatric treatments who might come from a variety of backgrounds, including children and adolescents, older adults, people with disabilities, and residents of both rural and urban areas. Intervention includes telepsychiatry (TP) services that are being implemented in the outpatient department (OPD), utilizing cutting-edge technologies to improve the delivery of mental healthcare, including big data, machine learning, AI, and virtual reality.		Outcomes assess the telepsychiatry services’ efficiency, dependability, affordability, and satisfaction from clients and providers. Additional results encompass the precision of diagnosis, clinical results, ease of use, financial consequences, and general effectiveness and quality of psychiatric care provided via telepsychiatry. The potential for technical developments like VR, ML, and AI to improve service delivery is also considered, as are implementation barriers.	Utilization of AI in Telepsychiatry In order to address treatment gaps, accessibility, diagnostic validity, and individual client preferences, the article highlights the growing use of telemedicine and telepsychiatry services in the outpatient department (OPD). Big data, AI, and other technological advancements are also incorporated to improve quality and efficacy. It addresses the clinical, administrative, technological, legal, and regulatory issues covered by telemedicine and telepsychiatry guidelines released by multiple nations, with the goal of offering TP services that give a level of care that is comparable to in-person consultations. Potential beyond the scope of AI The article highlights the possibility of considerably improving the general quality and effectiveness of TP services in OPDs by integrating cutting-edge technologies like virtual reality, machine learning, and AI into psychiatric healthcare delivery systems. Benefits of Telepsychiatry It discusses the advantages of telepsychiatry, such as its dependability, affordability, and happiness of clients and providers. It also raises the possibility that technical developments may make it possible for psychiatric specialists to give specialized care to people and locations that are underserved. Barriers to Telepsychiatry Implementation The article looks at a variety of obstacles to telepsychiatry adoption from the viewpoints of patients and providers, such as technological constraints, privacy issues, and socioeconomic issues. It concludes that these issues must be resolved in order to improve the acceptability and accessibility of TP services.
[22]	Digital Biomarker Representation Frailty Phenotypes: The Use of Machine Learning and Sensor-Based Sit-to-Stand Test Catherine Park et al. (2021)	The population consists of older people or veterans (over 65) who live in the community, are ambulatory, do not require assistance with standing or walking, and do not have any serious medical or mental health concerns. These people undergo evaluations for physical frailty phenotypes, with a special emphasis on the sit-to-stand test five times. The intervention consists of using a five-times sit-to-stand test. Machine learning approaches are integrated with sensor-based frailty modeling to discover the minimal number of sensor-derived features required for the identification of physical frailty and the three main frailty phenotypes (slowness, weakness, and weariness). Five wearable sensors were given to each participant so that their motions could be tracked throughout the test.		The main outcome is the detection of minimal features derived from sensors that accurately capture the frailty phenotypes of weakness, exhaustion, and slowness. Additionally, the performance of the frailty model is assessed using metrics like accuracy, sensitivity, specificity, and area under the curve (AUC). These results seek to confirm the efficacy of the sensor-based, machine learning-enhanced method for evaluating older persons’ physical fragility.	Research Aims This work addresses the shortcomings of conventional resource-intensive methods that are inappropriate for telehealth evaluations by investigating the use of machine learning in conjunction with frailty modeling to streamline and enhance frailty screening through sensor-based technologies. In order to facilitate the future design of low-cost, sensor-based technologies for remote physical frailty assessments via telemedicine, the research aims to determine the minimum number of sensor-derived features required to accurately identify physical frailty and key phenotypes (slowness, weakness, and exhaustion). Machine Learning Applications for Optimal Feature When combined with telemedicine frameworks, the use of machine learning for optimal feature selection in frailty modeling is an innovative technical technique that has the potential to greatly improve the quality of care and diagnostic precision for senior populations. The study’s conclusions may contribute to the creation of effective and user-friendly telemedicine solutions for frailty evaluation and remote monitoring, improving older individuals’ health outcomes and enabling prompt interventions. The talk highlights the possibilities for remote physical frailty assessments using sensor-based or sensor-less sit-to-stand tests made possible by machine learning. It also discusses the impact of this change toward telemedicine in post-COVID-19 healthcare on older people and their caregivers.
[23]	The Indian Health Service Primary Care-Based Teleophthalmology Program for Diabetic Eye Disease Surveillance and Management Stephanie J. Fonda et al. (2020)	American Indians and Alaska Natives (AI/AN) with diabetes make up the population. Historically, AI/AN have had lower rates of yearly diabetic retinopathy (DR) exams, which are the minimal required care. Additionally, this population tends to experience more diabetes complications. The Indian Health Service-Joslin Vision Network (IHS-JVN) Teleophthalmology Program is one type of intervention that has been put into place. It was started in 2000 with the goal of increasing AI/AN compliance with DR standards of care by using proven primary care-based telemedicine. With the use of this technology, DR severity can be remotely diagnosed. A report with management suggestions is also provided, and the patient’s primary care physician receives it back. The course complies with the Practice Guidelines for Ocular Telehealth-Diabetic Retinopathy published by the American Telemedicine Association (ATA).		Outcomes include better yearly rates of diabetic retinopathy exams, more patient knowledge, easier access to care, adherence to DR standards of care, and the teleophthalmology approach’s cost-effectiveness when compared to more conventional screening techniques. The program’s goal is to lower the risk of vision loss in AI/AN populations by providing comprehensive diabetes eye disease surveillance and management. The IHS-JVN has also made a significant contribution to studies on the use of diabetic eye care, cost-effectiveness, technological advancement, and the epidemiology of DR in AI/AN populations.	IHS-JVN Goals and Guidelines By utilizing telemedicine to enhance patient awareness and access to care, the IHS-JVN Teleophthalmology Program aims to raise the number of DR examinations for AI/AN with diabetes. In accordance with the ATA Practice Guidelines for Ocular Telehealth-Diabetic Retinopathy, the IHS-JVN offers remote diagnosis of DR severity and sends a report with therapy suggestions to the patient’s primary care physician. The IHS-JVN’s clinical process begins with the proactive recruitment of patients during primary care visits, signifying an integrated telemedicine approach to primary care and specialized care. The program’s usefulness in enhancing the standard of diabetic eye disease management among AI/AN populations has been demonstrated by its usage in studies on the use of diabetic eye care, cost-effectiveness, technological development, and DR epidemiology.
[24]	A comprehensive Review on Smart Healthcare: Applications, Paradigms, and Challenges with Case Studies Syed Saba Raoof et al. (2022)	The population comprises the general public with an emphasis on healthcare applications; it specifically targets people with particular healthcare needs, such as patients with chronic conditions, the elderly who need constant supervision, and people living in remote areas without easy access to medical facilities. Deep learning (DL) and the Internet of Things (IoT) are two smart healthcare technologies that are being used in intervention to improve patient monitoring, disease detection and diagnosis, emergency systems, and chronic disease self-management, among other aspects of healthcare. This covers a wide spectrum of applications, from sophisticated systems for deep learning algorithm-based disease diagnosis to wearable gadgets for tracking vital signs.		Improvements in early disease detection, improved diagnostic accuracy, better patient engagement and adherence to treatment plans, lower healthcare costs, and general improvements in patient outcomes and quality of life are examples of outcomes. Furthermore, these technologies can help alleviate healthcare disparities, particularly in underprivileged areas.	AI Smart Healthcare Applications Integration Outcomes Deep learning and IoT technology are being integrated to improve emergency response systems, patient monitoring, and illness identification in the healthcare industry. Smart healthcare monitoring systems that use sensor data and deep learning to anticipate heart illnesses are an example of how cutting-edge technologies can be applied to improve early intervention and diagnosis accuracy. The application of effective solutions, such as intelligent speech recognition technologies, for patients who are elderly or disabled, demonstrating how AI and IoT may support more individualized and accessible healthcare services. The use of deep learning and machine learning algorithms to analyze cardiac signals and provide real-time analytics highlights how AI can improve the accuracy and efficiency of heart disease detection. The use of wearable sensors and IoT devices in monitoring patients’ health conditions remotely, indicating the importance of technology in improving healthcare accessibility and enabling continuous patient care outside traditional settings.
[25]	Automated Diabetic Retinopathy Diagnosis for Improved Clinical Decision Support Boyle, J. et al. (2024)	Population comprises remote indigenous villages in Queensland and the Northern Territory. The majority of people in this population have diabetes and are at risk of developing diabetic retinopathy (DR), which is the main preventable cause of blindness in this population. Intervention consists of the deployment of a telemedicine platform powered by AI for eye screening services, which includes AI components for diagnosing diabetic retinopathy (DR) and detecting picture quality alerts. This service makes it easier to screen for and identify DR by putting patients in distant areas in contact with ophthalmologists in metropolitan areas.		The efficacy of the AI-powered telehealth platform in identifying diabetic retinopathy and evaluating the quality of images, as determined by prediction performance (specificity, sensitivity, and accuracy) in comparison to conventional ophthalmologist assessments. Improvements in the availability of specialized care, early identification of diabetic retinopathy, a decrease in pointless in-person consultations, and the possibility of more efficient management of eye health among isolated indigenous groups are among the outcomes.	Quality and Evaluation of AI/Telehealth eye-screening In order to reduce avoidable blindness due to diabetic retinopathy in remote indigenous communities, a telehealth-based eye screening service including AI-based picture quality and disease detection algorithms has been established. The telehealth platform’s AI components were evaluated for their prediction performance. It was found that the algorithm for detecting image quality was accurate 72% of the time at the individual image level and 85% at the patient level, while the algorithm for detecting retinopathy was accurate 85% of the time at the individual image level and 87% at the patient level. The potential for future service models utilizing AI to improve the caliber and accessibility of eye care services is demonstrated by the usage of the AI-based telehealth platform to supplement and support the decisions made by eye health assessment teams. In order to guide future teleophthalmology workflows incorporating AI components, the study aimed to evaluate the predictive effectiveness of the AI algorithms, which had been created on a different picture database, on retinal images obtained from remote indigenous communities. Given the indigenous ethnicity of the study’s patients, the AI’s performance in detecting diabetic retinopathy highlights the significance of high sensitivity in ensuring that patients with DR are not overlooked. It also demonstrates the AI’s effectiveness in a clinical setting and its comparability to other published efforts.
[26]	Image Quality Improvements of Hand-Held Ultrasound Devices With a Two-Stage Generative Adversarial Network Zhou, Z. et al. (2020)	Remote indigenous communities in Northern Territory and Queensland, primarily individuals affected by diabetes and at risk for diabetic retinopathy (DR), a leading cause of avoidable blindness among this population. Implementation of an AI-based telehealth platform for eye screening services, incorporating AI components for image quality alert detection and diabetic retinopathy (DR) diagnosis. This service connects metropolitan-based ophthalmologists with patients in remote locations to facilitate the screening and early detection of DR.		The effectiveness of the AI-based telehealth platform in detecting diabetic retinopathy and assessing image quality, as evaluated by its prediction performance (accuracy, sensitivity, and specificity) compared to standard ophthalmologist evaluations. Outcomes include improvements in accessibility to specialist care, early detection of diabetic retinopathy, reduction in unnecessary face-to-face consultations, and the potential for more effective management of eye health among remote indigenous populations.	AI and RPM Integration: The article discusses the integration of AI with remote patient monitoring (RPM) technologies to improve chronic disease management and patient outcomes. AI algorithms help analyze data collected from RPM devices to predict acute events and enable early intervention, thereby enhancing the quality of care for patients with chronic conditions. AI and RPM Integration In order to enhance the management of chronic diseases and patient outcomes, the article addresses the integration of AI with remote patient monitoring (RPM) technology. AI algorithms assist in the analysis of data gathered from RPM devices in order to forecast acute occurrences and provide early intervention, improving the standard of care for patients with long-term illnesses. Telemedicine for Chronic Disease Management The article emphasized that telemedicine systems can be used to give patients with chronic illnesses ongoing care by enabling routine monitoring without requiring in-person hospital visits. This strategy improves patient management and satisfaction, lowers hospital readmissions and ER visits, and aids in the early diagnosis of possible health problems. Barriers to Adoption The article addresses obstacles to the broad use of RPM and telemedicine technologies, such as technological difficulties, provider opposition, and problems with reimbursement and legal frameworks. According to the paper, removing these obstacles is essential to maximizing the benefits of RPM and telemedicine in terms of bettering patient outcomes and healthcare delivery. Patient Engagement and Education The importance of patient education and involvement to the efficient use of RPM and telemedicine technology are addressed. According to the article, better patient outcomes, more patient participation, and greater adherence to treatment programs can result from teaching patients about the advantages and appropriate usage of these tools.
[27]	Dermatology Image Quality Assessment (DIQA): Artificial intelligence to ensure the clinical utility of images for remote consultations and clinical trials Montilla, I.H. et al. (2023)	The population comprises those who use dermatological imaging in clinical practice and clinical studies for diagnosis, severity evaluation, and remote consultations. This includes individuals who need image-based assessments due to a variety of dermatological diseases. Dermatology Image Quality Assessment (DIQA), an AI-based tool, is used in the intervention to assess the quality of dermatological images and make sure they satisfy the minimal visual requirements required for clinical interpretation by physicians or algorithms. The purpose of this tool is to enhance the clinical value of photographs in clinical trials and remote consultations.		The main outcomes are an increase in the clinical value and quality of dermatological images used in clinical trials and remote consultations, as measured by the correlation between the quality scores predicted by AI and the quality scores observed by humans. The utilization of high-quality images can lead to secondary outcomes such as improved patient safety, diagnostic accuracy, and clinical process efficiency. Results could also include a possible decrease in clinical trial disruptions and patient safety issues related to low-quality photographs.	AI Quality, Implementation, and Effectiveness In order to evaluate and guarantee the quality of dermatological images—which are essential for diagnosis, severity evaluation, and efficient remote consultations in clinical practice and trials—the Dermatology Image Quality evaluation (DIQA) tool was developed and put into use. The application of non-expert observers to assess the visual quality of dermatological photographs according to particular standards, including focus, illumination, and distance to the lesion—all of which are critical in establishing the images’ therapeutic utility. In an effort to standardize and enhance the quality evaluation procedure, the AI technology is then utilized to forecast a quality score that represents the collective viewpoint of these observers. In order to maximize the prediction of image quality scores, multiple AI model configurations are experimented with, including transfer learning and training with diverse datasets. The efficacy of the AI in assessing picture quality pertinent to dermatological care is determined by measuring the models’ mean absolute error and correlation with human assessment. DIQA Implementation The DIQA tool has the potential to be used in clinical trials and remote consultations, especially in dermatology, where picture quality is crucial for precise diagnosis and treatment planning. This is a reflection of the growing use of AI technologies in telemedicine to improve patient safety and quality.
[28]	Global Burden of Cardiovascular Diseases and Risks, 1990–2022 Mensah, G.A. et al. A. et al. (2023)	The population is made up of people who have cardiovascular illnesses (CVD), which include people with rheumatic heart disease, ischemic heart disease, several types of stroke, hypertensive heart disease, and other circulatory and cardiovascular disorders. The study focuses on people worldwide, paying close attention to regional differences. The intervention is not really addressed because the study’s goal is to measure health loss resulting from hundreds of illnesses, accidents, and risk factors in order to improve health systems and eradicate inequities. Nonetheless, actions pertaining to medical care, public health campaigns, and legislative modifications with the intention of lessening the incidence and consequences of heart disease could be considered implicit interventions.		Primary outcomes include age-standardized death rates, prevalence rates, and disability-adjusted life years (DALYs) related to various cardiovascular illnesses. The study examines how these results have evolved over time and differ among nations and regions, demonstrating the efficacy of health policies and interventions in the management of CVD.	Results presented by the article No particular cases pertaining to AI/telemedicine and diagnosis/quality of treatment were immediately recognized based on the portions that were presented. Without focusing specifically on AI or telemedicine therapies, the publication mainly provides statistical data and trends about the regional and worldwide burdens of cardiovascular diseases.
[29]	Digital Health Applications to Establish a Remote Diagnosis of Orthopedic Knee Disorders: Scoping Review van Eijck et al., (2023)	Patients with a primary knee diagnosis in orthopedic surgery. Digital health and telemedicine applications in comparison with imaging or face-to-face contact between patients and physicians. Injury- and lifestyle-related factors were used.	Specificity and sensitivity analysis were made based on diagnoses by physicians and patients, the probability of having meniscus and screening for osteoarthritis.	There are only a limited number of available applications designed for remote diagnosis of knee disorders in orthopedic surgery. As of now, there is insufficient evidence to demonstrate that digital health applications can effectively aid either patients or orthopedic surgeons in establishing the primary diagnosis of knee disorders.	
[30]	Quality, Usability, and Effectiveness of mHealth Apps and the Role of Artificial Intelligence: Current Scenario and Challenges Deniz-Garcia, A. et al. (2023)	The study’s population includes patients with chronic, noncommunicable diseases (NCDs), such as cardiovascular disease, chronic respiratory disease, cancer, and diabetes. The study’s intervention includes mHealth apps designed for noncommunicable diseases (NCDs), and AI’s role is assessed as well.	The outcome of this study is the evaluation of the role of AI, and the quality, usability, and effectiveness of mHealth apps.	MHealth App Patient Utilization The usability of mHealth Apps is determined by various features, such as a simple and intuitive interface, clear graphical representations, easily understandable instructions, actionable tasks, minimal manual input requirements, provision of in-app educational content, and assistance with app utilization when necessary. However, the primary challenge lies in ensuring accessibility for all users. To assess the usability of mHealth apps, metrics such as MAUQ, SUS, and Questionnaire for User Satisfaction, and Post-Study System Usability assessments are commonly used. Goal setting, real-time feedback, rewards, use of data in social networking, easy data collection (by minimizing manual data entry), interactions with HCPs, social interaction and interactive communication among users and/or with the system, reminders, gamification, and journaling lead to behavioral changes with mHealth apps. Patient engagements are evaluated by number of logins, frequency of use, data entry, duration of use (total or by session), task completion, or self-reported use. Quality and Role of mHealth and AI The quality of mHealth apps should be evaluated by instruments such as MARS, ABACUS, HoN, mHealth Service Quality Scae, ORCHA, and Discern Instrument. WHO guidelines and legal requirements should be followed to ensure quality in the implementation of mHealth apps. AI plays an important role in disease diagnosis, monitoring, prevention and personalized medicine.	
[31]	Innovations in Research and Clinical Care Using Patient-Generated Health Data Heather S.L. Jim et al. (2020)	The study’s population includes patients with cancer. The intervention is patient-generated health data (PGHD) in oncology research and care.	The outcome is clinical monitoring, coupled with digital phenotyping (including biometrics, physical activity, diet, and functional status) using PGHD, has been identified to inform regulatory decisions, research, and cancer care delivery.	Benefits to PGHD in the patient population PGHD is being used to develop novel, data-driven methods for early detection and intervention in clinically critical events, such as toxicity and cancer progression. The use of PGHD may result in fewer hospital stays and ER visits. In order to manage symptoms, encourage healthy lifestyle choices, and improve drug adherence, it also makes it possible to have more intimate contact with patients outside of clinical settings. Challenges to PGHD Several technological, analytical, and procedural issues need to be resolved before PGHD can be effectively implemented in routine cancer research and therapy.	
[32]	Drug Allergy Labels Lost in Translation: From Patient to Charts and Backwards Allison Ramsey et al. (2021)	The population of this study are patients with drug hypersensitivity reaction.	n/a	Benefits to AI in Drug Labeling This is a prescriptive paper discussing how to record, label, and de-label adverse drug reactions using electronic health records, telemedicine, and electronic consultations. In order to improve accurate drug allergy labeling, the research recommends using AI and natural language processing in addition to ongoing education.	
[16]	Determinants for scalable adoption of autonomous AI in the detection of diabetic eye disease in diverse practice types: key best practices learned through collection of real-world data Goldstein et al., (2023)	The use of autonomous AI (Luminetics Core) to detect diabetic retinopathy at the point of care in various health centers across the Midwest, Southwest, Northeast, and West Coast (four healthcare centers).	The aggregated number of eye exams increased from 89 per month to 174 per month across all sites.	Successful of the utilization of fully autonomous AI was due to: (1) Inclusion of healthcare executives and clinical champions—administrative and physicians. (2) Availability of Health Center Resources (3) Clinical workflow that identify patient (pre-visit). LumineticsCore Exam Capture and Provider Consult (patient visit) and timely referral triage (post visit)	
[33]	Toward telemedical diagnostics—clinical evaluation of a robotic examination system for emergency patients Maximilian Berlet et al. (2024)	The study’s population includes 10 healthy volunteers and 10 physicians from a local emergency department. The intervention consists of auscultation, palpation, percussion, and telemedical examination using a robot to measure vital signs.		Among the outcomes are the patients’ acceptance of the employment of a robotic telemedicine technique to examine them.	This article meets inclusion criteria because it studies the use of telemedical robots to examine patients.
[34]	Ocular surface assessment in times of sanitary crisis: What lessons and solutions for the present and the future? Labetoulle et al. (2021)	n/a		n/a	Benefits of teleconsultation compared to tele-screening In contrast to telescreening alone, this research examines the use of true teleconsultation for remote ocular surface assessment. The authors propose using decision-making algorithms to assist optometrists in remotely screening their patients in order to determine the best time for follow-up consultations.
[35]	Implantable devices for heart failure monitoring Ijaz et al. (2021)	The population of this study is heart failure patients. The intervention is the use of CardioMEMS device.		n/a	Implantable Devices Impact Based on pilot research with 17 HF patients and the CHAMPION trial, this article describes the current role for Implantable Hemodynamic Monitors (IHM) in the care of HF patients. It also discusses the usage of various IHM devices and the evidence surrounding safety and efficacy. In general, CadioMEMS is safe.
[36]	No sonographer, no radiologist: New system for automatic prenatal detection of fetal biometry, fetal presentation, and placental location Arroyol et al. (2022)	The study’s population involves 58 pregnant women in Lima, Peru. The intervention includes the use of standardized volume sweep imaging (VSI) protocol based on external body landmarks to obtain imaging without an experienced sonographer and application of deep learning algorithm (U-Net).		In the study’s outcome, the U-net showed high accuracy in fetal presentation (cephalic vs. non-cephalic presentation) (100% agreement) and placenta position (placenta previa and other positions) (76% agreement) compared to standard of care ultrasound.	Impact on Accessibility and Quality The U-net increases access to prenatal care and ultrasound imaging in rural areas, however, the U-net has some limitations due to image size reduction. Benefits of AI The U-net showed 100% accuracy in fetal presentation and 76% accuracy in placenta position compared to standard of care ultrasound.

## References

[1] Nashwan A.J., Gharib S., Alhadidi M., El-Ashry A.M., Alamgir A., Al-Hassan M., Khedr M.A., Dawood S., Abufarsakh B. (2023). Harnessing Artificial Intelligence: Strategies for Mental Health Nurses in Optimizing Psychiatric Patient Care. Issues Ment. Health Nurs..

[2] Elbagoury B.M., Zaghow M., Salem A.-B.M., Schrader T. Mobile AI Stroke Health App: A Novel Mobile Intelligent Edge Computing Engine based on Deep Learning models for Stroke Prediction—Research and Industry Perspective. Proceedings of the 2021 IEEE 20th International Conference on Cognitive Informatics & Cognitive Computing (ICCI*CC).

[3] Upadhyaya P., Upadhyay S.K., Shrestha A., Shrestha N., Shrestha R., Khatri B., Pandey J., Subedi A., Dhungana S. Use of telemedicine and artificial intelligence in Eye and ENT: A boon for developing countries. Proceedings of the 2022 4th International Conference on Artificial Intelligence and Speech Technology (AIST).

[4] Sarapata G., Dushin Y., Morinan G., Ong J., Budhdeo S., Kainz B., O’Keeffe J. (2023). Video-Based Activity Recognition for Automated Motor Assessment of Parkinson’s Disease. IEEE J. Biomed. Health Informatics.

[5] D’Angelantonio E., Lucangeli L., Camomilla V., Pallotti A. Smart sock-based machine learning models development for patient screening. Proceedings of the 2022 IEEE International Workshop on Metrology for Industry 4.0 & IoT (MetroInd4.0&IoT).

[6] Hasumi E., Fujiu K., Nakamura K., Yumino D., Nishii N., Imai Y., Shoda M., Komuro I. (2023). A mutually communicable external system resource in remote monitoring for cardiovascular implantable electronic devices. Pacing Clin. Electrophysiol..

[7] Preity, Ranjan R., Verma K., Sahana B.C. A Computer-Aided Prediagnosis System for Health Prediction Based on Personal Health Data. Proceedings of the 2023 IEEE 12th International Conference on Communication Systems and Network Technologies (CSNT).

[8] Hameed K., Bajwa I.S., Ramzan S., Anwar W., Khan A. (2020). An Intelligent IoT Based Healthcare System Using Fuzzy Neural Networks. Sci. Program..

[9] Berger S.E., Agurto C., Cecchi G.A., Eyigoz E., Hershey B., Lechleiter K., Huynh D., McDonald M., Rogers J.L. The Impact of COVID-19 on Chronic Pain: Multidimensional Clustering Reveals Deep Insights into Spinal Cord Stimulation Patients. Proceedings of the 2023 IEEE International Conference on Digital Health (ICDH).

[10] Kiburg K.V., Turner A., He M. (2022). Telemedicine and delivery of ophthalmic care in rural and remote communities: Drawing from Australian experience. Clin. Exp. Ophthalmol..

[11] Grooby E., He J., Kiewsky J., Fattahi D., Zhou L., King A., Ramanathan A., Malhotra A., Dumont G.A., Marzbanrad F. (2021). Neonatal Heart and Lung Sound Quality Assessment for Robust Heart and Breathing Rate Estimation for Telehealth Applications. IEEE J. Biomed. Health Informatics.

[12] Ma J., Jia X., Li G., Guo D., Xi X., Zhou T., Liu J.-B., Zhang B. (2023). Development of 5G-based Remote Ultrasound Education: Current Status and Future Trends. Adv. Ultrasound Diagn. Ther..

[13] Liang W., Zhou C., Bai J., Zhang H., Jiang B., Wang J., Fu L., Long H., Huang X., Zhao J. (2024). Current advancements in therapeutic approaches in orthopedic surgery: A review of recent trends. Front. Bioeng. Biotechnol..

[14] Das S., Adhikary A., Laghari A.A., Mitra S. (2023). Eldo-care: EEG with Kinect sensor based telehealthcare for the disabled and the elderly. Neurosci. Informatics.

[15] Almadhi N.H., Dow E.R., Chan R.V.P., Alsulaiman S.M. (2022). Multimodal Imaging, Tele-Education, and Telemedicine in Retinopathy of Prematurity. Middle East Afr. J. Ophthalmol..

[16] Goldstein J., Weitzman D., Lemerond M., Jones A. (2023). Determinants for scalable adoption of autonomous AI in the detection of diabetic eye disease in diverse practice types: Key best practices learned through collection of real-world data. Front. Digit. Health.

[17] Liu H., Li R., Zhang Y., Zhang K., Yusufu M., Liu Y., Mou D., Chen X., Tian J., Li H. (2023). Economic evaluation of combined population-based screening for multiple blindness-causing eye diseases in China: A cost-effectiveness analysis. Lancet Glob. Health.

[18] Gupta P., Choudhury R., Kotwal A. (2023). Achieving health equity through healthcare technology: Perspective from India. J. Fam. Med. Prim. Care.

[19] Brady C.J., Garg S. (2020). Telemedicine for Age-Related Macular Degeneration. Telemed. e-Health.

[20] Grosjean S., Côté D., Grimes D., Mestre T. (2022). A digital companion (eCARE-PD platform) for people living with Parkinson: A co-design study. Int. J. Integr. Care.

[21] Vadlamani L.N., Sharma V., Emani A., Gowda M.R. (2020). Telepsychiatry and Outpatient Department Services. Indian J. Psychol. Med..

[22] Park C., Mishra R., Sharafkhaneh A., Bryant M.S., Nguyen C., Torres I., Naik A.D., Najafi B. (2021). Digital Biomarker Representing Frailty Phenotypes: The Use of Machine Learning and Sensor-Based Sit-to-Stand Test. Sensors.

[23] Fonda S.J., Bursell S.-E., Lewis D.G., Clary D., Shahon D., Horton M.B. (2020). The Indian Health Service Primary Care-Based Teleophthalmology Program for Diabetic Eye Disease Surveillance and Management. Telemed. e-Health.

[24] Raoof S.S., Durai M.A.S. (2022). A Comprehensive Review on Smart Health Care: Applications, Paradigms, and Challenges with Case Studies. Contrast Media Mol. Imaging.

[25] Boyle J., Vignarajan J., Saha S. (2024). Automated Diabetic Retinopathy Diagnosis for Improved Clinical Decision Support. Stud. Health Technol. Inform..

[26] Zhou Z., Wang Y., Guo Y., Qi Y., Yu J. (2020). Image Quality Improvement of Hand-Held Ultrasound Devices With a Two-Stage Generative Adversarial Network. IEEE Trans. Biomed. Eng..

[27] Montilla I.H., Mac Carthy T., Aguilar A., Medela A. (2023). Dermatology Image Quality Assessment (DIQA): Artificial intelligence to ensure the clinical utility of images for remote consultations and clinical trials. J. Am. Acad. Dermatol..

[28] Mensah G.A., Fuster V., Murray C.J., Roth G.A., Abate Y.H., Abbasian M., Abd-Allah F., Abdollahi A., Abdollahi M., Abdulah D.M. (2023). Global Burden of Cardiovascular Diseases and Risks, 1990–2022. J. Am. Coll. Cardiol..

[29] Van Eijck S., Janssen D., van der Steen M., Delvaux E., Hendriks H., Janssen R. (2022). Digital health applications to establish a remote diagnosis for orthopedic knee disorders: A scoping review. J. Med. Internet Res..

[30] Deniz-Garcia A., Fabelo H., Rodriguez-Almeida A.J., Zamora-Zamorano G., Castro-Fernandez M., Ruano M.d.P.A., Solvoll T., Granja C., Schopf T.R., Callico G.M. (2023). Quality, Usability, and Effectiveness of mHealth Apps and the Role of Artificial Intelligence: Current Scenario and Challenges. J. Med. Internet Res..

[31] Jim H.S.L., Hoogland A.I., Brownstein N.C., Barata A., Dicker A.P., Knoop H., Gonzalez B.D., Perkins R., Rollison D., Gilbert S.M. (2020). Innovations in research and clinical care using patient-generated health data. CA A Cancer J. Clin..

[32] Ramsey A., Macy E., Chiriac A.-M., Blumenthal K.G. (2021). Drug Allergy Labels Lost in Translation: From Patient to Charts and Backwards. J. Allergy Clin. Immunol. Pract..

[33] Berlet M., Fuchtmann J., Krumpholz R., Naceri A., Macari D., Jähne-Schon C., Haddadin S., Friess H., Feussner H., Wilhelm D. (2024). Toward telemedical diagnostics—Clinical evaluation of a robotic examination system for emergency patients. Digit. Health.

[34] Labetoulle M., Sahyoun M., Rousseau A., Baudouin C. (2021). Ocular surface assessment in times of sanitary crisis: What lessons and solutions for the present and the future?. Eur. J. Ophthalmol..

[35] Afari M.E., Syed W., Tsao L. (2018). Implantable devices for heart failure monitoring and therapy. Heart. Fail. Rev..

[36] Arroyo J., Marini T.J., Saavedra A.C., Toscano M., Baran T.M., Drennan K., Dozier A., Zhao Y.T., Egoavil M., Tamayo L. (2022). No sonographer, no radiologist: New system for automatic prenatal detection of fetal biometry, fetal presentation, and placental location. PLoS ONE.

